# Number of antenatal care utilization and associated factors among pregnant women in Ethiopia: zero-inflated Poisson regression of 2019 intermediate Ethiopian Demography Health Survey

**DOI:** 10.1186/s12978-022-01347-4

**Published:** 2022-02-05

**Authors:** Mastewal Arefaynie, Bereket Kefale, Melaku Yalew, Bezawit Adane, Reta Dewau, Yitayish Damtie

**Affiliations:** 1 Department of Reproductive and Family Health, School of Public Health, College of Medicine and Health Sciences, Wollo University, PO Box 1145, Dessie, Ethiopia; 2 Department of Epidemiology and Biostatistics, School of Public Health, College of Medicine and Health Sciences, Wollo University, Dessie, Ethiopia

**Keywords:** Number, Antenatal care, Zero-inflated Poisson, Ethiopia

## Abstract

**Background:**

The frequency of antenatal care utilization enhances the effectiveness of the maternal health programs to maternal and child health. The aim of the study was to determine the number of antenatal care and associated factors in Ethiopia by using 2019 intermediate EDHS.

**Methods:**

Secondary data analysis was done on 2019 intermediate EDHS. A total of 3916.6 weighted pregnant women were included in the analysis. Zero-inflated Poisson regression analysis was done by Stata version 14.0. Incident rate ratio and odds ratio with a 95% confidence interval were used to show the strength and direction of the association.

**Result:**

About one thousand six hundred eighty eight (43.11%) women were attending four and more antenatal care during current pregnancy. Attending primary education (IRR = 1.115, 95% CI: 1.061, 1.172), secondary education (IRR = 1.211, 95% CI: 1.131, 1.297) and higher education (IRR = 1.274, 95% CI: 1.177, 1.378), reside in poorer household wealth index (IRR = 1.074, 95% CI: 1.01, 1.152), middle household wealth index (IRR = 1.095, 95% CI: 1.018, 1.178), rich household wealth index (IRR = 1.129, 95% CI: 1.05, 1.212) and richer household wealth index (IRR = 1.186, 95% CI: 1.089, 1.29) increases the number of antenatal care utilization. The frequency of antenatal care was less likely become zero among women attending primary (AOR = 0.434, 95% CI: 0.346, 0.545), secondary (AOR = 0.113, 95% CI: 0.053, 0.24), higher educational level (AOR = 0.052, 95% CI: 0.007, 0.367) in the inflated part.

**Conclusion:**

The number of antenatal care utilization is low in Ethiopia. Being rural, poorest household index, uneducated and single were factors associated with low number of antenatal care and not attending antenatal care at all. Improving educational coverage and wealth status of women is important to increase the coverage and frequency of antenatal care.

## Plain language summary

Antenatal care is among the most effective interventions to mitigate maternal mortality and morbidity. It is an entry point for delivery care, postnatal care and child immunization**.** This study was conducted to determine the frequency and associated factors of antenatal care utilization in Ethiopia by using 2019 intermediate Ethiopian Demography Health Survey.

A cross-sectional study design using secondary data from 2019 intermediate Ethiopian demography and health survey was conducted. 3917 weighted women were included in the study. Recoding, variable generation, labeling and analysis were done by using STATA/SE version 14.0.

The objective of this study was to identify the determinants of frequency of antenatal care visit in Ethiopia by using zero inflated Poisson regression.

In this study 74.38% of women attend antenatal care at least once during their current pregnancy. Only 41.8% of women use WHO recommended number of antenatal care.

Conclusion: maternal age, residence, educational status, household wealth index, religion and region show significant association with the frequency of antenatal care utilization. Advocacy and behavioral change communication should be area of concern for different organizations that are working on antenatal care especially for rural, poor and uneducated women through mass campaign, community dialoging and enhance the effectiveness of health extension programs.

## Introduction

Maternal mortality is global public health problem [1–8]. The problem is disproportionally high in developing countries including Ethiopia [2, 3, 8]. Pregnant women suffer from direct and indirect pregnancy related complications [2, 9–14]. Reproductive health is a global agendum for the last 50 years [15–18]. Despites the global efforts, reproductive health problems of women are not addressed [4, 6, 7, 9, 12, 19–22].

Antenatal care is among the most effective interventions to mitigate maternal and child mortality and morbidity [23–25]. It is an entry point for delivery care, postnatal care and child immunization. It also makes link between the health provider and the clint for further interventions [25–29]. During ANC pregnancy related complications, pre-existing health conditions are screened, diagnosed and appropriate interventions are delivered for pregnant women. Behavioral change communication on personal hygiene, utilization of available services and interventions are provided for the women and the family at large [23, 25, 27, 30–32].

Now a day’s ANC address a wide range services including, identifying threats during the prenatal period, birth preparedness and complication readiness, family planning, child feeding options and nutritional counseling during pregnancy and after birth [22, 28, 29, 31, 33–35]. WHO guide line recommends at least four ANC for normal pregnancy and extra visit for women with complications. The first visit is recommended in the first trimester which is predicted to screen and treat anemia, syphilis, HIV testing and counseling (HTC) and screen for risk factors and medical conditions. The second, third and fourth visits are scheduled at 24–28, 32 and 36 weeks, respectively to monitor fetal and maternal conditions [34].

In Ethiopia, maternal mortality ratio is high (412 per 10,000 life birth) [36]. The country has reproductive strategy for the last 20 years. ANC services are available to the country side and included in health extension package. ANC utilization is increased through time. But it still low [37].

In Ethiopia, different researches have been done on prevalence and/or factors associated with ANC utilization [35, 38–49]. Maternal educational status, age, residence, accessing radio, wealth index, pregnancy status, number of children, accessibility of health facilities, occupation and religion are determinant factors identified by scholars [50–63]. But, all the studies were done at local level with small sample size. There were studies in the Ethiopia by using Ethiopian demography and health surve-2016 by using logistic regression. However, it results loss of information, due to count nature of the outcome variable. More over recent national representative evidence is scarce. As a result in this study we account methodological limitation of previous studies by using count data analysis using recent national data. So the aim of this study was to determine the frequency and associated factors of antenatal care utilization in Ethiopia by using 2019 intermediate Ethiopian Demography Health Survey.

## Method

### Study setting and period

The study was conducted in Ethiopia, which is located in the North-eastern (horn of) Africa, lies between 3° and 15° North latitude and 33° 48° and East longitudes. This study used the intermediate EDHS 2019 dataset which was conducted by the Central Statistical Agency in collaboration with the federal Ministry of Health (FMoH) and the Ethiopian Public Health Institute. Data were accessed from their URL: www.dhsprogram.com by contacting them through personal accounts after justifying the reason for requesting the data. Then reviewing the account permission was given via the email. A cross-sectional study design using secondary data from 2019 intermediate Ethiopian demography and health survey was conducted.

### Sampling procedure

**Fig. 1:**
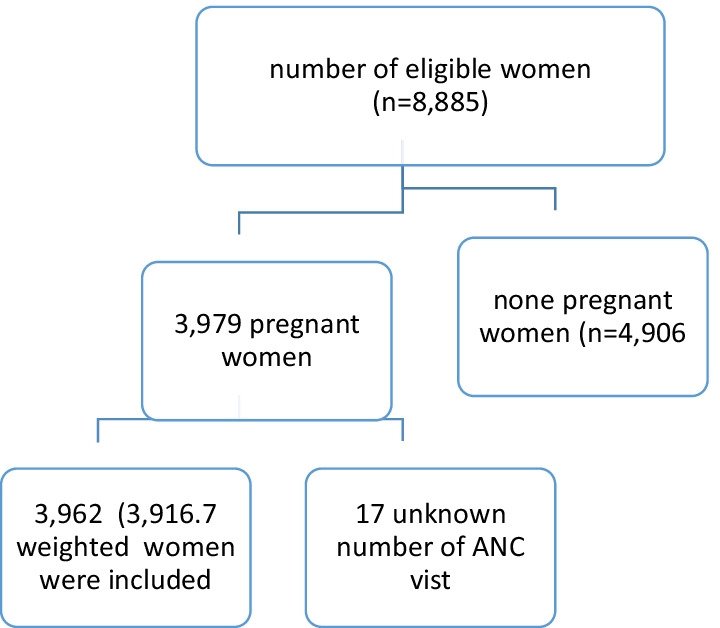
Sampling and exclusion procedures to identify the final sample size in intermediate 2019 EDHS

The intermediate EDHS 2019 sample was stratified and selected in two stages. In the first stage, stratification was conducted by region and then each region stratified as urban and rural, yielding 21 sampling strata. A total of 305 (94 urban 211 rural) enumeration areas (EAs) were selected with probability proportional to EA size in each sampling stratum. In the second stage households were selected proportionally from each EA by using systematic sampling method.3, 916.7 weighted women were included in the analysis (Fig. 1).

### Study variables

The outcome variable of this study was the number of ANC visits during last pregnancy. The independent variables include; women’s age, religion, current marital status, residence, educational level, household wealth index, region and number of children.

### Variable measurement

The number of antenatal care was measured as account data between 0 and 20. The regions were categorized in to three as urban (Addis Ababa and Dire Dawa), developed (Tigray, Amhara, Oromia and South Nations Nationalities people) and the rest (Afar, Somali, Gambella and Benishangul Gumz) were leveled as developing regions.

### Data processing and analysis

Data cleaning was conducted to check for the consistency with the EMDHS 2019 descriptive report. Recoding, variable generation, labeling and analysis were done by using STATA/SE version 14.0. Since ANC follow up (dependent variable) is a non-negative integer, most of the recent thinking in the field has used the Poisson regression model as a starting point. To run Poisson regression, mean and variance should be equal. In the current case the mean and the variance were 2.89 and 5.33 respectively. So the assumption is violated. That is the data were over dispersed. To handle over-dispersion and excess zeros in the data, we have considered zero inflated Poisson models, the extension of Poisson regression to have precise result [50].

$$\mathrm{P }(Yi = yi) =\left\{\begin{array}{c}\pi i+(1 - \pi i) exp (-\mu i) , if yi = 0\\ \left(1 - \pi i\right)exp \left(-y\mu i!i\right)\mu y ii, if yi= 1, 2, 3, . . \end{array}\right. 0 \le \pi i \le 1.$$

The analysis was done for both count part and the zero inflated part. Finally incident rate ratio and odds ratio were presented with 95% CI. Statistical significance was determined at a P value of less than 0.05.

## Results

From weighted 3916.7 pregnant women, 1688.3(43.11%) women use four and more antenatal care during current pregnancy. About 1003.5 (25.62%) women do not attend antenatal care during pregnancy. The mean and the variance of observations are 2.89 and 5.33 respectively (Table1).

**Table 1:** Number of women experiencing antenatal care in Ethiopia, 2019

Number of ANC visit	Count	Present
0	1003.5	25.62
1	130.24	3.33
2	293.38	7.49
3	801.16	20.46
4	919.83	23.49
5	404.93	10.34
6	223.69	5.71
7	65.82	1.68
8	42.37	1.07
9	17.08	0.44
10 +	14.64	0.37
Mean	2.89	
Variance	5.33	
Skewness	0.7	
Kurtosis	5.21	
Minimum	0	
Maximum	20	
Total observation	3916.7	

### Selection of model

Poisson regression, negative binomial regression and zero-inflated Poisson regression are tested to select model for analysis. Zero-inflated model has good log likelihood test (− 5692.033) than the two models. The Vuong test (< 0.001) indicates there is statistical difference between Poisson regression and zero-inflated Poisson regression (Table 2).

**Table 2:** Model selection to analysis number of antenatal care utilization in Ethiopia, 2019

Model	Log likelihood	Tests
Poisson	− 9201.6678	
Negative binomial	− 8634.555	alpha (< 0.001)
Zero-inflated Poisson	− 5692.033	Vuong test (< 0.001)

### Magnitude of ANC utilization among pregnant women in Ethiopia, 2019

The lowest mean numbers of ANC visits were observed from 45 to 49 age group women (1.94), while the highest visits were observed from 30 to 34 age group women (3.03) and 25–29 years old women (3.07). There was a significant difference on the mean number of ANC visits among some age groups. A significantly high mean numbers ANC visits were recorded in Orthodox religion followers (3.69). As educational level increases the mean numbers of ANC visits were significantly increased. The lowest mean was observed in uneducated women (2.18) and the highest was among women who attended higher educational level (5.02).

The lowest mean numbers of ANC visits were recorded among women who lived in the poorest household wealth index (1.63). The highest numbers of ANC visits were observed in urban dweller women (4.2). There is significant difference on the mean numbers of ANC visits across regions. The highest mean was observed in women who lived in urban regions (4.02) and the lowest was among women who lived in developing regions (2.15) (Table3).

**Table 3:** Number of antenatal care services utilization by socio-demographic characteristics of pregnant women in Ethiopia, 2019

Variable	Number	Percent	Mean (95% CI)
*Age*			
15–19	227.1	5.8	2.4 (2.15, 2.66)
20–24	767.2	19.6	2.96 (2.81, 3.11)
25–29	1190.3	30.4	3.07 (2.94, 3.19)
30–34	796.4	20.3	3.03 (2.86, 3.2)
35–39	589.1	15	2.79 (2.59, 2.98)
40–44	257.4	6.6	2.46 (2.16, 2.76)
45–49	89.2	2.3	1.94 (1.5, 2.38)
*Religion*			
Orthodox	1434.8	36.6	3.69 (3.56, 3.82)
Protestant	1082.2	27.6	2.72 (2.58, 2.86)
Muslim	1336	34.1	2.48 (2.38, 2.59)
Others*	63.6	1.6	1.63 (1.23, 2.03)
*Educational status*			
No education	2010.4	51.3	2.18 (2.08, 2.27)
Primary	1410.9	36	3.25 (3.13, 3.36)
Secondary	342.8	8.8	4.35 (4.13, 4.57)
Higher	152.6	3.9	5.02 (4.72, 5.31)
*Household wealth index*			
Poorest	823.4	21	1.63 (1.52, 1.74)
Poorer	821.6	20.9	2.62 (2.47, 2.77)
Middle	761.3	19.4	2.89 (2.73, 3.05)
Richer	703.1	17.9	3.27 (3.11, 3.43)
Richest	807.2	20.9	4.44 (4.29, 4.59)
*Marital status*			
Never married	20.8	0.5	2.41 (1.57, 3.25)
Married	3675.8	93.9	2.9 (2.82, 2.97)
Widowed/ divorced	220	5.6	2.92 (2.64, 3.2)
*Number of living children*			
0	39.1	1	2.24 (1.64, 0.85)
1–2	1634.6	41.7	3.43 (3.32, 3.54)
3–4	1071.5	27.4	2.78 (2.65, 2.92)
5 and above	1171.5	29.9	2.27 (2.14, 2.39)
*Residence*			
Urban	1020	26	4.2 (4.04, 4.35)
Rural	2896.7	74	2.46 (2.39,2.54)
*Region*			
Urban	157.3	4	4.02 (3.84, 4.2)
Developing	331.3	8.5	2.15 (2.05, 2.26)
Developed	3428.1	87.5	2.98 (2.88, 3.08)

*Catholic and traditional religion follower

### Factors associated with frequency of antenatal care

In the Poisson model, maternal age, residence, educational status, household wealth index, religion and region shows significant association with the frequency of antenatal care utilization.

The frequency of antenatal visit was 1.17 (IRR = 1.74, 95% CI: 1.052, 1.31) and 1.23 (IRR = 1.233, 95% CI: 1.075, 1.414) times higher among 30–34 years and 40–44 years women than 15–19 years women respectively.

The number of antenatal care visit was 12.7% (IRR = 0.873, 95% CI: 0.826, 0.924), 5.3% (IRR = 0.947, 95% CI: 0.903, 0.994) times more among Orthodox followers than Protestant and Muslim religious followers respectively.

Women attending primary education (IRR = 1.115, 95% CI: 1.061, 1.172), secondary education (IRR = 1.211, 95% CI: 1.131, 1.297) and higher education (IRR = 1.274, 95% CI: 1.177, 1.378) had high number of antenatal care visit than uneducated women. Women reside in poorer household wealth index (IRR = 1.074, 95% CI: 1.01, 1.152), middle household wealth index (IRR = 1.095, 95% CI: 1.018, 1.178), rich household wealth index (IRR = 1.129, 95% CI: 1.05, 1.212) and richer household wealth index (IRR = 1.186, 95% CI: 1.089, 1.29) had significantly high frequency of antenatal care. Urban dweller women had (IRR = 1.096, 95% CI: 1.028, 1.169) significantly high number of antenatal care follow up than rural dwellers.

In the zero inflated model, maternal age, marital status, educational status, household wealth index and religion shows significant association with antenatal care service utilization uptake becomes zero.

The number of antenatal care was 47.5%, 49.3% and 43.7% less likely became zero among 25–29 years old women (AOR = 0.525, 95% CI: 0.337, 0.816), 30–34 years old women (AOR = 0.507, 95% CI: 0.313, 0.819) and 35–39 years old women (AOR = 0.563, 95% CI: 0.34, 0.934) when compared with 15–19 years old women. The frequency of antenatal care was 56.6%, 88.7% and 94.8% less likely become zero among women attending primary (AOR = 0.434 95% CI: 0.346, 0.545), secondary (AOR = 0.113, 95% CI: 0.053, 0.24) and higher educational level (AOR = 0.052, 95%CI: 0.007, 0.367) than non-educated women respectively.

The number of antenatal care was 2.3 and 2.5 times more become zero among Protestant (AOR = 2.342, 95% CI: 1.749, 3.136) and Muslim religious followers (AOR = 2.512, 95% CI: 1.865, 3.284) than orthodox followers respectively (Table 4).

**Table 4:** Factors associated with ANC service utilization among pregnant women in Ethiopia, 2019

Variable	IRR	Inflated part AOR
*Age*		
15–19	1	1
20–24	1.059 (0.958, 1.171)	0.914 (0.601, 1.39)
25–29	1.096 (0.991, 1.213)	0.525 (0.337, 0.816)**
30–34	1.174 (1.052, 1.31)*	0.507 (0.313, 0.819)**
35–39	1.171 (1.041, 1.317)*	0.563 (0.34, 0.934)**
40–44	1.233 (1.075, 1.414)*	0.787 (0.449, 1.366)
45–49	1.162 (0.958, 1.41)	1.084 (0.554, 2.119)
*Religion*		
Orthodox	1	1
Protestant	0.873 (0.826, 0.924)*	2.342 (1.749, 3.136)**
Muslim	0.947 (0.903, 0.994)*	2.512 (1.865, 3.284)**
Others	0.701 (0.568, 0.865)*	2.732 (1.392, 5.36)**
*Educational status*		
No education	1	1
Primary	1.115 (1.061, 1.172)*	0.434 (0.346, 0.545)**
Secondary	1.211 (1.131, 1.297)*	0.113 (0.053, 0.24)**
Higher	1.274 (1.177, 1.378)*	0.052 (0.007, 0.367)**
*Household wealth index*		
Poorest	1	1
Poorer	1.074 (1.01, 1.152)*	0.447 (0.349, 0.572)**
Middle	1.095 (1.018, 1.178)*	0.415 (0.313, 0.551)**
Richer	1.129 (1.05, 1.212)*	0.278 (0.201, 0.384)**
Richest	1.186 (1.089, 1.29)*	0.196 (0.126, 0.307)**
*Marital status*		
Never married	1	1
Married	1.107 (0.853,1.437)	0.219 (0.079, 0.609)**
Widowed/ divorced	1.078 (0.823, 1.413)	0.215 (0.073, 0.633)**
*Number of living children*		
0	1	1
1–2	1.055 (0.867, 1.285)	0.423 (0.203, 1.879)
3–4	1.012 (0.827,1.239)	0.636 (0.297,1.362)
5 and above	1.005 (0.818, 1.235)	0.645 (0.296, 1.404)
*Residence*		
Urban	1.096 (1.028, 1.169)*	0.883 (0.627, 1.245)
Rural	1	1
*Region*		
Urban	1	1
Developing	0.904 (0.851, 0.962)*	1.305 (0.957, 1.777)
Developed	0.957 (0.902, 1.015)	1.196 (0.854, 1.674)

*Has significant association in IRR and ** on AOR

## Discussion

The objective of this study was to identify the determinants of frequency of antenatal care visit in Ethiopia by using zero inflated Poisson regression.

In this study 74.38% of women attend antenatal care at least once during their current pregnancy. The finding is consistent with researches in Afghanistan (69.3%) [64], Southern Ethiopia (76.2%) [40], Zambia (69%) [65], Nepal (76.0%) [66]. The finding is higher than the study in Benishangul Gumuz Region, Ethiopia (37.7%) [62], Nigeria (65.1%) [67], Eastern Ethiopia (53.6%) [59]. The finding was lower than studies in Southwestern Ethiopia (91.9) [68], Ghana (98.3%) [69], Pakistan (83.5%) [70], Guinea (80.3%) [55]. The finding indicates, the country needs more effort to improve the coverage of ANC. Since all pregnant women should attend ANC at least once during pregnancy.

In this study, only 41.8% of women use WHO recommended number of antenatal care. The finding is lower than studies in Rwanda (54%) [71], sub-Saharan countries (58.53) [61], India (51.7%) [72], Nigeria (56.2%) [67] and Pakistan (57.3) [70]. The finding is higher than findings in Bangladesh (32%) [73], Eastern Ethiopia (15.3%) [59] and Zambia (29%) [65]. The difference might be due to study population coverage, study setting and time. The finding shows there is a gap between the country health transformation plan (95%) and the situation in the ground (41.8%). It indicates different stake holders should work to increase the frequency of ANC to reduce maternal and child mortality.

Women in the middle age group have high frequency of antenatal care utilization. The probability of antenatal care utilization not zero is lower in this group of women. The result is similar with findings in Ethiopia [51], Uganda [74], Bangladesh [75], East African Countries [60], Nigeria [76, 77]. Middle age women might experiences previous pregnancy related complications that make them conscious to use maternal health services [54, 78, 79].

The frequency of antenatal care visit was high in urban dwellers than rural residents. The finding is in line with previous researches [55, 56, 64, 80]. Urban residence reduces distance to get services [81–84]. Rural dwellers might face transportation problem to access the service [78, 79, 85, 86]. Moreover, urban dwellers might have mass media accesses on importance of antenatal care [61, 87–89]. Since 74% of (Table 3) women resided in rural part of the country, more effort is expected from the government to increase the number of ANC at national level.

The frequency of antenatal care increases as the educational status of the women was increased. The odds of not attending antenatal care service were reduced when educational status is increased. The finding was supported by previous researches in Western Ethiopia [62], Ethiopia [56], rural Ethiopia [51], Kenya [90], Guinea [55, 91], Afghanistan [64], Angola [54], East African countries [60], India [92, 93], Ghana [94], Nepal [95], Nigeria [96]. Educated women might empowered to get services [97–102], education make women to have decision making [103, 104]. Moreover educated women have knowledge on danger sign [105–108]. Educated women might have awareness on the advantage of antenatal care different services provided in the service delivery points for the fetus and herself [53, 109–111].

As the household wealth index increases the frequency of antenatal care service utilization is increased and decreases the probability of not taking antenatal care services. The estimated IRR and OR, indicates that the probability no antenatal care take and the frequency of ANC visits increased with increasing household wealth status. The result was consistent with other findings in Western Ethiopia [62], Nepal [112], Guinea [55, 113], Angola [54], Nigeria [67], India [72], Ghana [69] Bangladesh [73, 102]. It might be due to women who belong to rich household usually have higher [114, 115] educational status, access to mass media [55, 116–118], and an ability to spend more money to take frequent ANC visits compared to women from poorer families [119–121].

There was religious variation on the frequency and utilization of antenatal care. Orthodox religion followers have high number of antenatal care utilization than Protestant and Muslim religious followers. Studies suggest there is religious variation on utilization and frequency antenatal care [75, 122, 123]. Further qualitative research might be needed to have more information on the effect of religion on utilization of ANC.

The odds of not antenatal care were less in married and widowed/divorced women than single women. The result is consistent with findings in Rwanda [83], Debre Berhan [124], Benin [125], Middle and low income countries [126]. Married women might get partner support to attend antenatal care [55, 127, 128]. Moreover, single women might face stigma to use antenatal care services by the community and health care workers [129–131].

Women who lived in developing regions had low frequency of antenatal care utilization than women who resided in urban areas. The finding is agreed with previous researches in different parts of Africa [54, 56, 132, 133]. There might be differences in socio-cultural, power relationships, accessibility of service in different parts of the country.

The result of this study was more representative than other studies and the model considered different levels of analysis as the outcome was count data. Despite this strength, the result may be prone to recall bias because the data were collected from a history of the event and some variables were missed since the data set was intermediate. Due to the nature of the data set, quality related data, previous exposure variables, paternal variables were not included.

## Conclusion

Utilization of minimum number of antenatal care utilization is low in Ethiopia. Being in middle age group, urban residence, increased educational level, improved household wealth status, Orthodox religious follower increases the number of antenatal care utilization.

Being middle age group, married, increased educational status, improved household wealth status; Orthodox religion follower decreases the probability of not attending antenatal care. Advocacy and behavioral change communication should be area of concern for different organizations that are working on antenatal care especially for rural, poor and uneducated women through mass campaign, community dialoging and enhance the effectiveness of health extension programs. Further, qualitative research would be needed to get detail information how religion was affect the frequency of ANC.

## Data Availability

The datasets used and/or analysed during this study are available from the corresponding author on reasonable request.

## Abbreviations

CSA: Central Statistics Agency
EA: Enumeration area
